# Brain-Derived Neurotrophic Factor Trafficking via the Regulated Secretory Pathway: Mechanisms and Disease Implications

**DOI:** 10.1007/s12035-025-05381-8

**Published:** 2025-11-19

**Authors:** Sharday N. Ewell

**Affiliations:** Department of Biology, University of Mississippi, University (Oxford), Oxford, MS 38677 USA

**Keywords:** BDNF, Trans-Golgi network, Regulated secretory pathway, Intracellular trafficking, Neurodevelopmental disorders, Neurodegenerative disorders

## Abstract

Brain-derived neurotrophic factor (BDNF) plays an essential role in neuronal development and synaptic plasticity. BDNF’s role in neuronal function is dependent on its trafficking through the regulated secretory pathway (RSP). This review provides a synthesis of the molecular mechanisms underlying anterograde BDNF trafficking with an emphasis on the role of sorting receptors (e.g., sortilin, carboxypeptidase E), motor proteins (e.g., kinesin), and scaffolding proteins (e.g., huntingtin, huntingtin-associated protein-1 1, dynactin). Despite major advances in elucidating the molecular players involved in BDNF trafficking, many regulators, such as adaptor proteins, ADP ribosylation factor GTPases, granins, and myosins, remain poorly characterized. However, given that impaired BDNF trafficking has been implicated in multiple neurodegenerative, neuropsychiatric, and neurodevelopmental disorders, understanding the trafficking of BDNF in full not only uncovers fundamental aspects of BDNF function but also reveals potential therapeutic targets for neurological disorders that are associated with impaired BDNF trafficking. This review summarizes current knowledge of anterograde BDNF trafficking from biosynthesis to regulated exocytosis and highlights its importance for neuronal function and human disease.

## Introduction

Brain-derived neurotrophic factor (BDNF) is a member of the neurotrophin family that is widely expressed throughout the brain and plays a key role in neurodevelopment, dendritic differentiation, neurogenesis, synaptic plasticity, and learning and memory [1–10]. BDNF’s functions are tightly dependent on its subcellular localization and regulated secretion. Specifically, BDNF is trafficked via the regulated secretory pathway (RSP) where it is sorted into secretory granules at the trans-Golgi network (TGN) and released at the plasma membrane in a Ca^2+^-dependent manner in response to neuronal activity.

The trafficking of BDNF through the RSP is proposed to be essential for its function, as the dysregulation of BDNF trafficking has been implicated in numerous neuronal disorders, as disruptions in sorting receptors, motor protein complexes, or scaffold proteins can impair BDNF targeting to the RSP and reduce activity-dependent secretion [3, 4, 11–22].

Despite its critical role in neuronal function, the trafficking of BDNF through the RSP has received relatively little focused attention. This review discusses the molecular components involved in the biogenesis, sorting, transport, and secretion of BDNF, focusing on proteins that target BDNF to the RSP and facilitate its trafficking. It is important to note that much of what is known about regulated secretory trafficking has been elucidated from heterologous cell systems (e.g., HEK293) or neurosecretory cells (e.g., PC12 cells). While these models are useful for describing the molecular mechanisms underlying regulated secretory trafficking, they may not be generalizable to neurons. Therefore, interpretations from these models are presented with caution and, where possible, contrasted with evidence from neuronal cultures or in vivo studies. In contrast to prior reviews, this article synthesizes the findings across multiple studies to provide a comprehensive description of the regulators of BDNF trafficking in the regulated secretory pathway and links these mechanisms to major depressive disorder, schizophrenia, autism spectrum disorders, Huntington’s disease, and Alzheimer’s disease. Finally, this review also highlights unresolved mechanistic questions and conceptual gaps for future investigation.

## Biogenesis, Maturation, and Transport of BDNF-Containing Secretory Granules

Vesicular trafficking is an important process that underlies neuronal function as it serves to deliver critical cargo, including neurotrophic factors (e.g., BDNF and neurotrophin-3) [23–25], neuropeptides (e.g., neuropeptide Y) [26], receptors (e.g., δ-opioid receptor 1 (DOR1) and β2 adrenergic receptor (AR)), ion channels (e.g., voltage-gated calcium channel α2δ1 subunit, voltage-gated potassium channel Kvβ2 subunit), and preassembled receptor complexes to the plasma membrane [27]. As such, secretory trafficking not only plays a role in synaptic function, but it also underlies neuronal differentiation and spine morphogenesis, with previous studies demonstrating that the manipulation of proteins involved in regulated secretory trafficking [11, 28, 29] or manipulations that block secretory trafficking [30] result in decreased dendritic outgrowth and impaired neuronal differentiation and spine morphogenesis.

Transmembrane and secreted proteins are first synthesized in the endoplasmic reticulum (ER) and are trafficked via transport vesicles to the Golgi. At the Golgi, these cargo molecules are then sorted and packaged into vesicles that are destined for specific trafficking pathways at the TGN [31]. Some vesicles traffic to endosomes or lysosomes, while others are destined for exocytosis at the plasma membrane via constitutive secretion. The constitutive secretory pathway is responsible for the synthesis of new membrane, the trafficking of plasma membrane proteins, and the stimulus-independent secretion of extracellular molecules and extracellular matrix proteins [32]. Alternatively, cargo can be sorted into the regulated secretory pathway. This pathway is responsible for the calcium-dependent release of neuropeptides, neurotrophic factors, granins, neurohormones, and transmembrane proteins [33] (Table 1). Preferential sorting to the constitutive pathway or the regulated secretory pathway has primarily been characterized in neurosecretory cell models (e.g., PC12 cells). In neurons, sorting into each pathway is more difficult to characterize, and a limited number of studies have quantified constitutive release of neurotrophic factors. For example, it has been demonstrated that nerve growth factor (NGF), a member of the neurotrophin family, sorts to both the constitutive and regulated secretory pathway [34–36]. Therefore, strong conclusions regarding neurotrophin sorting in neurons should be made with caution.

**Table 1:** Comparison of trafficking via the RSP to the constitutive pathway

Feature	RSP	Constitutive pathway
Release	Activity-dependent, Ca^2+^	Continuous
Cargo	BDNF NGF Neuropeptides Hormones	Membrane proteins NGF Neurotrophin-4 (NT-4)

However, multiple studies have demonstrated that BDNF is preferentially trafficked via the regulated secretory pathway in neurons [37–39]. Consistent with this, endogenous BDNF colocalizes with secretory granule markers such as furin in neurons, is preferentially sorted to the RSP, and is released in an activity-dependent manner [23–25].

In the RSP, the BDNF precursor proBDNF is synthesized in the endoplasmic reticulum and trafficked to the Golgi. At the TGN, it interacts with sorting receptors such as sortilin and carboxypeptidase E for packaging into immature secretory granules (ISGs) [9, 40, 41]. These ISGs undergo homotypic fusion and become progressively acidic, promoting cargo condensation and creating an environment favorable for proteolytic processing [42, 43]. Within ISGs, proBDNF is cleaved by peptidases such as furin and pro-convertase (PC) 2 to generate the mature, active form of BDNF [35, 44, 45]. This proteolytic step influences trafficking fate as uncleaved proBDNF is diverted to the constitutive pathway while mature BDNF is preferentially sorted into the RSP for activity-dependent release [44].

Additional proteins and lipids within ISGs (e.g., mannose-6-phosphate receptor, carboxypeptidase D, and Golgi-associated γ-ear-containing ARF-binding protein) are selectively removed via clathrin-coated vesicles and recycled back to the TGN via retrograde trafficking or to another compartment (e.g., endosomes) via anterograde trafficking. These remodeling steps facilitate the development of mature secretory granules (MSGs). BDNF-containing MSGs are then trafficked to the plasma membrane through the recruitment of motor proteins such as kinesins [46]. These motor proteins are recruited to the BDNF-containing MSGs through interactions with proteins such as carboxypeptidase E (CPE), dynactin, and huntingtin-associated protein 1 (HAP1) [46].

**Fig. 1:**
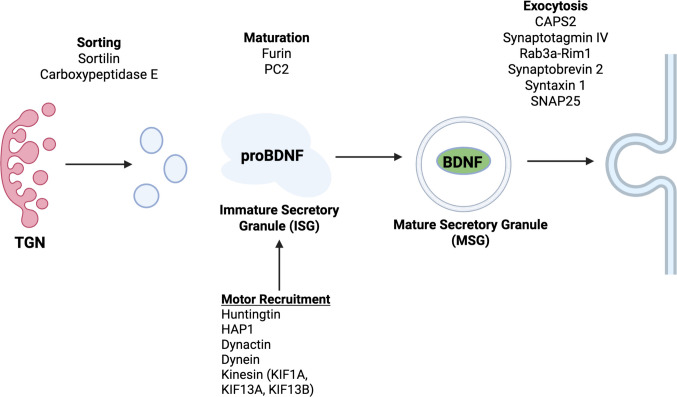
Biogenesis, maturation, and transport of BDNF-containing secretory granules. Created with BioRender. Ewell, S. (2025) https://BioRender.com/ii4ktk0

Once near the plasma membrane, the regulated exocytosis of BDNF occurs following a rise in calcium levels that is triggered by a depolarization-dependent activation of voltage-gated calcium channels or intracellular calcium release. This final step is regulated by the calcium sensors calcium-dependent activator protein for secretion 2 (CAPS2) and synaptotagmins [47–49] as well as SNARE complex proteins (Fig. 1).

While the trafficking via the RSP involves the coordinated action of multiple proteins, BDNF is unique in that it traffics to both axons and dendrites, suggesting differential sorting mechanisms, motor recruitment, and vesicle fusion machinery [23, 48, 50]. However, to date, the regulators of BDNF trafficking are not well characterized. The following sections will discuss the sorting receptors, scaffolding proteins, and motor proteins identified as regulators of BDNF trafficking.

## Regulators of BDNF Trafficking

### Sorting Receptors

In neurons, BDNF is preferentially sorted to the RSP at the TGN [35, 50]. One of the processes that contributes to this sorting is the interaction of BDNF with sorting receptors located at the TGN [51, 52]. Specifically, BDNF contains sorting motifs in the pro domain region and mature region that can interact with the sorting receptors CPE and sortilin [9, 41, 53].

#### Sortilin

Sortilin is a member of the Vps10-domain family of sorting receptors that is abundantly expressed in neurons throughout the central and peripheral nervous system [9, 29, 54]. Approximately 90% of sortilin is localized to the TGN, immature secretory granules, and endosomes, indicating a multifunctional role in intracellular trafficking [9, 55]. Sortilin has been demonstrated to regulate the trafficking of NGF [9, 56]. Specifically, sortilin and receptor p75 bind to the prodomain of NGF to facilitate pro-NGF-induced cell death. Based on the sequence homology of NGF and BDNF prodomains and their role in facilitating trafficking from the Golgi, Chen and colleagues [9] hypothesized that sortilin interacts with the prodomain of BDNF to facilitate its sorting to the RSP. It was observed that sortilin colocalizes with BDNF in neurosecretory cells and primary cultured hippocampal neurons. Additionally, the expression of a mutated form of BDNF that contained a methionine substitution in the prodomain region resulted in reduced association with sortilin. It was also observed that the expression of truncated sortilin and the small interfering RNA (siRNA)-mediated knockdown of sortilin in primary neurons resulted in the missorting of BDNF to the constitutive pathway, further supporting the finding that sortilin regulates the sorting of BDNF to the RSP [9].

Sortilin also has a cytoplasmic tail that contains sorting motifs that are essential for the binding of adaptor proteins, Golgi-associated γ-ear-containing ARF-binding proteins (GGAs), and other proteins essential for the trafficking of sortilin [55]. However, to date, it is unknown which molecules are required to direct sortilin itself into the regulated secretory pathway, with previous studies demonstrating that sortilin can sort cargo to the constitutive pathway, the regulated secretory pathway, and the endosomal pathway [9, 57, 58]. For example, sortilin has been demonstrated to sort proBDNF to the regulated secretory pathway in neurons [9]. However, when proBDNF is produced in excess, sortilin will target proBDNF to the endolysosomal pathway [54]. While the mechanisms that direct sortilin to each of these pathways are not well elucidated, recent work suggests that the binding of sortilin to cargo induces conformational changes that direct sortilin to specific trafficking pathways [55, 59]. Alternatively, before interaction with sortilin, cargo destined for different pathways may localize to different regions of the Golgi [60]. Consistent with this, Chen and colleagues demonstrated that cargo sorting occurs in the early Golgi, and this sorting results in distinct regions that will generate carriers for cargo to be trafficked via the constitutive pathway, regulated secretory pathway, or the lysosomal pathway [55, 60]. Because of this early sorting, sortilin binds the cargo and other accessory proteins (e.g., clathrin, APs) specific to each pathway.

#### Carboxypeptidase E

CPE, also known as carboxypeptidase H and enkephalin convertase, is an enzyme that localizes to secretory granules and is required for the biosynthesis of peptide hormones and neuropeptides. CPE activates neuropeptides by acting as an exopeptidase and removing C-terminal residues from precursor neuropeptides [61]. CPE has also been identified as a sorting receptor that facilitates the sorting of proneuropeptides (e.g., proenkephalin, proinsulin, and proneurotrophins) to regulated secretory granules [41, 46, 50, 51, 61, 62]. CPE targets cargo to the RSP through interaction with two acidic residues and two hydrophobic residues on the sorting signal domain [61]. As a result of this interaction, the cargo is segregated at the TGN and sorted to the RSP [50].

Given that CPE interacts with sorting signal motifs on pro-opiomelanocortin (POMC) and proinsulin [62, 63], Lou and colleagues [41] hypothesized that proBDNF would be sorted to the RSP via a similar mechanism. Indeed, X-ray analysis of the crystal structure of BDNF identified a sorting motif. Additionally, a mutation of this motif resulted in the increased constitutive secretion of BDNF. Furthermore, it was shown that BDNF is constitutively secreted in CPE knockout mice [42]. Taken together, these data demonstrate that BDNF can be sorted to the RSP through its interaction with CPE.

### Accessory Proteins

The TGN serves as a critical sorting station for newly synthesized proteins [64, 65]. At the TGN, protein cargo is sorted into distinct vesicles that are destined for various locations, including endosomal compartments and the plasma membrane [9, 40, 41]. To ensure fidelity in sorting, neurons use sorting receptors and an elaborate cargo sorting machinery. Specifically, cargo adaptor proteins, ADP ribosylation factors (Arfs), coat proteins, and accessory proteins are essential for sorting cargo at the TGN [64, 66]. To date, only one accessory protein has been demonstrated to facilitate BDNF trafficking.

#### Huntingtin-Associated Protein 1

HAP1 was the first huntingtin (htt) binding partner to be identified [18]. In neurons, HAP1 is distributed throughout the brain and is essential for neuronal function [18]. HAP1 localizes to the cytoplasm, the TGN, vesicles, and microtubules, indicating a role in mediating anterograde intracellular transport [17–19, 67–69]. Further supporting the role of HAP1 in anterograde transport is the interaction of HAP1 with other trafficking-related molecules [18, 70]. HAP1 has been demonstrated to interact with the p150^Glued^ subunit of dynactin, a motor-coordinating protein that facilitates the recruitment of dynein and kinesin to secretory granules [15, 16, 18, 70]. HAP1 also colocalizes and interacts with kinesin light chain [71]. Furthermore, the suppression of HAP1 function in live neurons resulted in the inhibition of kinesin-dependent vesicular trafficking [71].

HAP1 mediates the axonal transport of BDNF, with prior studies demonstrating that HAP1^−/−^ mice display aberrant BDNF trafficking as indicated by alterations in BDNF distribution throughout the neuron and decreased activity-dependent release of proBDNF [18, 19]. Studies to further characterize the role of HAP1 in anterograde BDNF trafficking have uncovered that HAP1 interacts with the dynactin complex that facilitates the recruitment of kinesin and dynein motor proteins to secretory granules (described below) [17].

In studies that further elucidated the mechanism underlying HAP1’s regulation of BDNF transport, it was found that endogenous HAP1 colocalizes with proBDNF and sortilin in cultured cortical neurons. Furthermore, mapping studies using pulldown and competition assays demonstrated that HAP1 interacts with sortilin and proBDNF [72]. Additionally, it was found that these three proteins form a complex that is required for proBDNF trafficking from the Golgi, facilitates mature BDNF release through cleavage by furin (an endopeptidase associated with BDNF-containing secretory granules), and prevents proBDNF degradation. Taken together, these data highlight the role of HAP1 in BDNF trafficking through its interactions with proBDNF, dynactin, huntingtin, and sortilin.

### Scaffolding Complexes

The recruitment of molecular motors to BDNF-containing secretory granules is mediated through the coordinated action of scaffolding proteins. These proteins are the components of larger complexes that possess the ability to bind to cargo sorting receptors (e.g., sortilin), molecular motors (e.g., kinesin and dynein), and other proteins required for trafficking, such as adaptor proteins and GTPases [73].

#### Huntingtin

Htt is a large protein that is distributed throughout the cytoplasm, where it associates with vesicles, microtubules, molecular motors, and other proteins associated with vesicular trafficking, suggesting a potential role for htt in regulating intracellular trafficking [17, 73]. Consistent with this, it has been observed that htt associates with HAP1. HAP1 is a protein that associates with the motor coordinating protein dynactin and facilitates the recruitment of motor proteins (e.g., kinesin and dynein) to secretory granules [15, 17]. Caviston and colleagues [74] demonstrated that huntingtin and dynein are present on vesicles and directly interact with one another. Furthermore, it was observed that dynein is responsible for the localization of htt in the cell. Taken together, these findings indicate that htt acts with dynein to facilitate vesicular transport [74]. Further pointing to a role for huntingtin in regulating intracellular trafficking, Gunawardena and colleagues [75] and Szebenyi and colleagues [76] demonstrated that the expression of mutant forms of huntingtin or the loss of huntingtin resulted in impaired axonal transport in neurons.

#### Dynactin

The action of motor proteins can be enhanced through their association with a second binding domain, protein, or protein complex [77]. The dynactin complex localizes to secretory granules and facilitates the bidirectional transport of secretory granules through its association with the motor proteins dynein, kinesin-2, and kinesin-3 [46, 78–80]. By recruiting these motor proteins, dynactin mediates retrograde and anterograde BDNF trafficking.

Dynactin is a motor coordinator that recruits motor proteins that mediate anterograde or retrograde trafficking to BDNF-containing secretory granules in neurons [17, 46, 78]. The disruption of this complex has been shown to disrupt the trafficking of BDNF. Using live cell imaging to investigate secretory granule trafficking in neurons, Kwinter and colleagues [78] observed that the addition of a dynactin disruptor resulted in decreased secretory granule transport. Similarly, in cellular and mouse models of Huntington’s disease, it was observed that the huntingtin/HAP1/dynactin complex was altered. Furthermore, the alteration of this complex resulted in reduced BDNF transport and subsequent release [17]. Finally, Park and colleagues [46] used live cell imaging of primary cultured hippocampal neurons to investigate the role of the sorting receptor CPE in the bidirectional transport of BDNF. Interestingly, it was demonstrated that CPE interacts with dynactin. Furthermore, dynactin interacted with KIF3A, KIF1A, and dynein [46]. Taken together, these data suggest that BDNF transport is facilitated through dynactin.

### Motor Proteins

The transport of secretory granules to the synaptic terminal requires the action of molecular motors. There are three superfamilies of motor proteins, myosins, kinesins, and dynein, that interact with actin filaments and microtubules to transport cargo [77, 80, 81]. Myosins associate with actin filaments to facilitate anterograde transport [82]. Conversely, kinesin and dynein motors associate with microtubules [83]. Dynein motors facilitate the retrograde transport of cargo in axons and transport in both directions (anterograde and retrograde) in dendrites [83], while kinesins facilitate anterograde trafficking. The localization of motor proteins to subcellular compartments is critical for their function in facilitating the trafficking of specific cargo. Studies visualizing the localization of fluorescently tagged, constitutively active members of the kinesin family in primary cultured hippocampal neurons have demonstrated that kinesins localize preferentially to axons or axons and dendrites [84].

In vitro studies conducted by Vershinin et al. [85] demonstrated that multiple motors are associated with secretory granule cargo. Furthermore, while multiple motors may associate with secretory granules, in vivo studies indicate that only a single motor is actively engaged at a given time [86, 87]. To date, dynein and members of the kinesin-3 family have been directly implicated in BDNF trafficking. This section will discuss the motor proteins implicated in anterograde BDNF trafficking and their localization within the neuron.

#### Kinesins

The kinesin superfamily of molecular motors plays a critical role in the anterograde transport of BDNF-containing granules along microtubules [81]. There are 15 kinesin families that are classified as kinesin 1 to kinesin 14B [81]. Of these, members of the kinesin-3 family have been identified as molecular motors that facilitate the anterograde trafficking of BDNF. KIF1A is essential for the axonal transport of dense core vesicles [88–90]. Using live cell imaging, Lo et al. [88] demonstrated that fluorescently tagged chromogranin A and BDNF move with fluorescently tagged KIF1A in both anterograde and retrograde directions in primary culture hippocampal neurons, suggesting a role for KIF1A in BDNF transport. Reduction of KIF1A expression via shRNA resulted in significantly reduced transport of BDNF-containing secretory granules, further indicating a role in BDNF trafficking [88]. Similarly, Park and colleagues [46] showed that in addition to its role as a sorting receptor, CPE also interacts with dynactin, KIF1A, and KIF3A, a member of the kinesin-2 family, to mediate anterograde BDNF trafficking. CPE also interacts with dynein for retrograde trafficking.

BDNF is known to undergo compartment-specific trafficking, with some granules transported to dendrites or axons [23, 48, 91]. Recent work by Montgomery and colleagues [92] suggests a role for KIF13A and KIF13B, also members of the kinesin-3 family, in this targeting. In primary culture hippocampal neurons, these motors localize to the TGN where KIF13A exclusively plays a role in dendrite-specific trafficking, while KIF13B facilitates dendritic-specific and axonal-specific transport [90, 92].

### Proteins That Facilitate Granule Priming, Docking, and Fusion

Given the role of BDNF in neuronal function, more work has been done to identify the proteins that facilitate the exocytosis of BDNF-containing secretory granules. This section will discuss the proteins involved in BDNF secretion: Rab3a-Rim1, CAPS2, synaptotagmins, and SNARE complex proteins.

### Rab3a-Rim1

Ras-associated binding (Rab) proteins are the largest family of small GTPases with over 60 identified protein isoforms in the human genome [93]. Many of these proteins function in the regulation of vesicle fusion and exocytosis through their association with vesicular membranes and the recruitment of effector proteins such as rab-interacting molecule (RIM) [93–95]. The Rab3s are the predominant Rab proteins located in the brain and include Rab3A, Rab3B, Rab3C, and Rab3D. Rab3A, Rab3B, and Rab3C facilitate the fusion of BDNF-containing granules to the plasma membrane [93, 95]. Consistent with this, Persoon and colleagues [95] found that the inactivation of all Rab3 genes in neurons resulted in the ablation of secretory granule fusion that was restored following the re-expression of Rab3a, suggesting a key role for Rab3a in the fusion process. Similarly, dense core vesicle (DCV) exocytosis was undetectable in neurons lacking Rim1 but was restored following the expression of full-length Rim1 and a short N-terminal RIM1 fragment that contained a Rab3 binding domain [95].

### CAPS2

CAPS2 is a member of the CAPS protein family that is associated with secretory granules containing BDNF and plays a role in their exocytosis [11, 47]. Structurally, CAPS2 contains both a dynactin 1 interaction domain, suggesting that it may coordinate motor complex recruitment, and the Munc13-1 homologous domain, which is implicated in vesicle priming.

Genetic disruption of CAPS2 in mice results in reduced BDNF secretion and synaptic plasticity deficits. Previous studies have demonstrated that CAPS2 knockout mice have impaired BDNF secretion [11], and mice expressing an alternatively spliced CAPS2 variant that lacked the region involved in binding to dynactin 1 also resulted in altered BDNF secretion [11]. Altogether, these data indicate that CAPS2 serves as a priming factor that couples exocytosis to cytoskeletal transport.

### Synaptotagmin 4

The synaptotagmin family of proteins is comprised of 17 members that interact with SNARE proteins (see “SNARE Proteins”) in a Ca^2+^-dependent manner to facilitate the exocytosis of synaptic vesicles and dense core vesicles [95]. Synaptotagmin 4 (syt4) has been implicated in the regulation of BDNF exocytosis. Syt4 can bind to SNARE proteins in a Ca^2+^-independent manner but fails to bind SNAREs or penetrate membranes in the presence of Ca^2+^ [96]. Syt4 localizes with and traffics BDNF in primary culture hippocampal neurons [49]. Furthermore, in hippocampal neurons where syt4 levels were reduced, there was an increased rate of exocytosis. On the other hand, in neurons where syt4 was overexpressed, a decrease in exocytosis was observed [49]. Collectively, these data suggest that syt4 plays a role in limiting the exocytosis of BDNF-containing secretory granules.

In addition to its role in facilitating the exocytosis of BDNF-containing vesicles as a calcium sensor, recent studies have demonstrated that syt4 has a broader role in the targeting and release of BDNF-containing vesicles. Specifically, syt4 has been implicated in the targeting of BDNF-containing vesicles to axons and dendrites [97]. In dendrites, syt4 and the motor protein KIF1A are trafficked together along microtubules into dendritic spines in an activity-dependent manner, allowing for the positioning of vesicles near synaptic sites for exocytosis [98]. In hippocampal neurons, the phosphorylation of syt4 at residue S135 by c-Jun N-terminal kinases (JNK) destabilizes its interaction with the motor protein KIF1A. This triggers the transition of dense core vesicles from microtubule-dependent transport to actin-dependent capture at dendritic spines specifically [99].

Syt4 also directly interacts with the neuronal scaffold protein ankyrin repeat-rich membrane spanning (ARMS) to negatively regulate BDNF secretion [100, 101]. Consistent with this, López-Benito and colleagues [100] demonstrated that the knockdown of ARMS in the cortex and hippocampus resulted in increased BDNF secretion. Furthermore, it was demonstrated that ARMS regulates syt4 levels, with knockdown of ARMS levels resulting in decreased endogenous syt4 levels in cortical neurons [100]. Taken together, these studies demonstrate that in addition to its role as a Ca^2+^ sensor, syt4 functions as a scaffolding protein that serves to facilitate the localization of BDNF-containing vesicles to specific locations in the neuron and their activity-dependent release.

Syt4 may also regulate other aspects of BDNF trafficking. For example, hippocampal neurons from syt4 knockout mice were observed to have structural defects in the TGN [102]. Furthermore, in studies in neurosecretory PC-12 cells, it was observed that syt4 facilitates ISG maturation [103]. This points to a potential role of syt4 in regulating the biogenesis and maturation of BDNF-containing secretory granules, but this has not been tested directly in neurons.

### SNARE Proteins

SNARE complexes are a core component of the presynaptic release machinery that allows synaptic vesicles and secretory granules to fuse with the plasma membrane [104–107]. These complexes include SNARE proteins, synaptobrevin (syb2), SNAP25, and syntaxin 1 [106]. In addition to facilitating the release of synaptic vesicles, Shimojo and colleagues [106] demonstrated that syb2, SNAP25, and SNAP47 facilitate BDNF release in cortical neurons. It was also demonstrated that syb2 and SNAP25 colocalized with CAPS2, a calcium sensor associated with BDNF-containing secretory granules [107]. Taken together, these data indicate that SNARE complex proteins facilitate BDNF secretion.

## Future Directions

Prior research has revealed several proteins that serve to sort BDNF into secretory granules or serve as part of the molecular motor complex that transports BDNF-containing granules down microtubule tracks in the neuron. CPE and sortilin act as sorting receptors for mature BDNF and proBDNF, respectively. Furthermore, the anterograde trafficking of BDNF-containing granules is facilitated through HAP1, dynactin, huntingtin, and kinesin [17, 19, 78, 88] (Fig. 1). However, despite the progress made in enhancing our understanding of the molecular mechanisms that regulate BDNF secretory trafficking, multiple areas for future research remain. For example, the role of granins in BDNF sorting, the recruitment of adaptor proteins and Arf GTPases, and the mechanisms underlying motor protein specificity remain incompletely characterized. However, recent advances in imaging (e.g., live cell imaging with BDNF-GFP or BDNF-pHluorin) [108, 109], genetic manipulation (e.g., CRISPR-Cas9), and proteomics (e.g., protein–protein binding assays) may be promising methods that can be used to address these questions that were previously inaccessible. A summary of these questions can be found in Table 2.

**Table 2:** Summary of outstanding questions regarding BDNF trafficking in neurons

Mechanistic area	Outstanding questions	Current knowledge
Co-trafficking and cargo interactions	Do granins promote BDNF aggregation and facilitate interaction with CPE or sortilin? How do co-packaged cargoes (e.g., neuropeptides, receptors, granins) influence BDNF maturation, sorting, and secretion?	SGIII interacts with CPE at the TGN [40, 110]; BDNF colocalizes with SGII, but its role is unclear [111]; BDNF is co-packaged with neuropeptides and granins; vesicle composition can be modified by regulators such as syt4 [103]
Adaptor proteins, Arf GTPases, and coat proteins	Which Arfs, GAPs, and GEFs regulate BDNF trafficking? Which adaptor proteins (AP-1, AP-3, AP-4, GGAs) associate with BDNF-containing granules?	Arf1 and Arf6 regulate maturation/exocytosis in other systems [42, 112–118]; ADAP1 colocalizes with BDNF-containing vesicles and interacts with Arf6, KIF13B, and AP-3 [119, 120]
Molecular motor recruitment	Which kinesins beyond KIF1A and KIF13A/B transport BDNF vesicles? How are dendritic vs axonal transport fates determined?	KIF13A/B interacts with AP-1 with KIF13A granules targeted to the axon and KIF13B granules targeted to dendrites [92]; CPE recruits dynactin for motor binding [46]; KIF5 implicated in DCV transport but direct link to BDNF not shown [121]
Myosins and vesicle fusion	Which myosins regulate approach, docking, priming, and fusion of BDNF vesicles?	Myosins Va, 1c, and 1e implicated in approach/tethering; myosins VI and II implicated in fusion; no direct evidence for BDNF [82]
Cell type–specific regulation	Does BDNF sorting differ across neuronal subtypes?	Differential levels of trafficking proteins across subtypes [122, 123]; differences in mechanisms remain undefined
Synaptic vs extrasynaptic release	What determines whether BDNF vesicles fuse at synaptic or extrasynaptic sites?	BDNF vesicle release at both sites [124, 125]; tethering/docking factors may bias localization but remains uncharacterized

### The Role of Other Cargo Sorted with BDNF

BDNF-containing vesicles also carry additional cargoes such as neuropeptides and high molecular weight neurotransmitters [126]. However, to date, it is unknown if these molecules are co-released with BDNF or if they modulate the sorting and maturation of BDNF-containing immature secretory granules or release [126]. For example, in addition to CPE and sortilin, the sorting of cargo into secretory granules is also mediated by the granin family of proteins (e.g., chromogranin A, chromogranin B, secretogranin II, secretogranin III). Granins facilitate cargo sorting through the aggregation of cargo proteins (e.g., prohormones, proneuropeptides) into secretory granules. However, aggregation alone is not sufficient for sorting into the RSP [110, 127, 128]. Rather, this aggregation is an essential step that promotes the interaction of cargo with sorting receptors (e.g., CPE and sortilin) at the TGN membrane [51]. For example, secretogranin III (SGIII) interacts with cholesterol located at the TGN. As a result of this interaction, SGIII serves as a sorting receptor for chromogranin A (ChrA) [51, 109]. SGIII and ChrA can then work together to target proteins to the regulated secretory pathway [51, 111, 129, 130]. Furthermore, it has been demonstrated that SGIII can also interact directly with CPE [40, 110]. In line with this, mice expressing defective CPE exhibited elevated levels of SGIII, which further suggests a sorting function for SGIII. Previous studies have demonstrated that BDNF colocalizes with SGII in primary cultured hippocampal neurons [40]. However, the role of SGII in BDNF sorting is not well defined [111]. Given their known role in cargo aggregation for neuropeptides, future studies should investigate whether granins similarly promote the aggregation of BDNF. This may serve to enhance its interaction with CPE or sortilin, further promoting the preferential sorting of BDNF into the RSP. Future studies should test if co-packaged molecules regulate BDNF vesicle fusion dynamics or activity-dependent release.

### The Role of Adaptor Proteins, Arf GTPases, and Coat Proteins in BDNF Sorting and Motor Protein Recruitment

Motor proteins are recruited to secretory granules by scaffolding proteins (e.g., huntingtin), which are, in turn, recruited by Arfs, Arf GTPase-activating proteins (GAPs), Arf guanine nucleotide exchange factors (GEFs), adaptor proteins, and coat proteins. Arfs are members of the Ras superfamily of small GTPases and are comprised of six isoforms. While all Arfs have been implicated in the regulation of secretory or endosomal trafficking, Arf1 and Arf6 are the best characterized [112, 131–135]. Arf1 localizes to the Golgi and immature secretory granules where it recruits the coat protein clathrin as well as the adaptor protein (AP)−1 and facilitates secretory granule maturation [42, 43, 112–114] while Arf6 localizes primarily to endosomal compartments and the plasma membrane where it regulates endocytosis, exocytosis of regulated secretory granules, and receptor recycling [134, 136–142]. Arf6 has also been demonstrated to localize to immature secretory granules in chromaffin cells [115] and associates with immature secretory granule membranes in vitro [116], suggesting a potential role for Arf6 in the regulated secretory pathway in neurons.

In regulated secretory trafficking, Arfs recruit cargo adaptor proteins (e.g., APs and GGAs) to the TGN and other secretory compartments to facilitate the budding of vesicles [64]. As a GTPase, Arfs cycle between a GTP and GDP-bound state that is facilitated by GEFs and GAPs [117, 134, 143]. Through this cycling, Arfs recruit coat proteins (e.g., clathrin), coat adaptor proteins, cargoes, lipid-modifying enzymes, and cytoskeletal regulators to budding vesicles [112]. To date, it is unknown which Arf is associated with BDNF-containing secretory granules. Furthermore, the Arf GAPs and GEFs that are associated with this process are not well characterized. However, Arf GAP with dual PH domain 1 (ADAP1) may play a role in BDNF trafficking. ADAP1 is expressed throughout the cortex, including the hippocampus, and has been demonstrated to regulate dendritic differentiation and spine morphogenesis in primary culture hippocampal neurons [119, 120]. ADAP1’s role in regulating dendritic differentiation may be due to its role in regulating vesicular trafficking. Consistent with this, ADAP1 displays a punctate staining pattern in primary culture hippocampal neurons and interacts with proteins that play a role in trafficking, including Arf6 [116], the kinesin motor protein KIF13B [144, 145], and the adaptor protein AP-3 [117]. Furthermore, Ewell [118] demonstrated that endogenous ADAP1 localizes with markers of regulated secretory granules such as chromogranin B, furin, and AP-3 in primary culture hippocampal neurons. Endogenous BDNF has also been demonstrated to colocalize with ADAP1 in both the soma and the dendrites of primary culture hippocampal neurons, suggesting a role for ADAP1 in regulating BDNF trafficking [118]. Future studies should use genetic manipulation (e.g., siRNA knockdown), immunocytochemistry, and live imaging to continue to investigate which Arfs, GAPs, and GEFs regulate the trafficking of BDNF.

Adaptor proteins are recruited to vesicle membranes by Arf GTP [119, 140]. There are four adaptor complexes (AP-1, AP-2, AP-3, and AP-4) that recruit coat proteins (e.g., clathrin) and other effector proteins to secretory granules [120]. For example, previous studies have demonstrated that AP-3 [117], an adaptor protein that regulates trafficking to endosomal compartments, is regulated by Arf1 [146–150]. While AP-3 has been identified as an adaptor protein that is associated with BDNF-containing secretory granules, to date, it is not known if other adaptor proteins are associated with BDNF-containing granules. Future studies should continue to investigate which adaptor proteins regulate anterograde BDNF trafficking.

### The Mechanisms Underlying Molecular Motor Recruitment to BDNF-Containing Secretory Granules

The kinesin superfamily of proteins comprises 40 proteins that display cargo and compartment specificity [81]. While studies have identified KIF1A, KIF13A, and KIF13B as mediators of anterograde BDNF transport, the full spectrum of kinesins associated with BDNF-containing granules remains incompletely defined. For example, KIF5, a member of the kinesin-1 family, has been implicated in the transport of dense core vesicles [121] and interacts with motor adaptors such as HAP1 and dynactin [151, 152]. However, its direct association with BDNF has not yet been demonstrated. Future studies should test whether this interaction contributes to compartment-specific BDNF transport.

It has been extensively documented that BDNF displays differential trafficking in dendrites and axons [23, 48, 91]. Therefore, a current area of study has been to investigate the molecular mechanisms that underlie these differences. The kinesins KIF13A and K13B, members of the kinesin-3 family, have been demonstrated to mediate dendritic and axonal transport [92, 153–155]. However, *how* these proteins are recruited to BDNF-containing granules that will be differentially transported is not well characterized. Recently, Montgomery and colleagues [92] demonstrated that KIF13A and KIF13B interact with the adaptor protein AP-1 at the TGN, and the disruption of this interaction resulted in impaired trafficking in both dendrites and axons [92]. Similarly, Park and colleagues [46] demonstrated that in addition to functioning as a sorting receptor, CPE also facilitates motor recruitment by binding dynactin, which in turn recruits KIF1A or KIF3A for anterograde BDNF trafficking or dynein for retrograde trafficking. These findings suggest that adaptors such as AP-1 and CPE play a role in recruiting molecular motors to BDNF-containing secretory granules in axons and dendrites.

Other proteins, such as ADAP1, which has been shown to interact with KIF13B, remain unexplored in the context of BDNF trafficking. Additionally, ArfGTPases may play a role in the recruitment of molecular motors, but their roles are not well understood. Future studies should aim to characterize the full complement of adaptor and motor proteins that govern BDNF trafficking across neuronal compartments.

Collectively, future studies that uncover the mechanisms underlying motor recruitment may characterize how BDNF-containing vesicles are targeted to axons or dendrites while also providing a foundation for testing whether these mechanisms differ across neuronal subtypes. Notably, neuronal subtypes have been shown to express different complements of trafficking machinery [122, 123]. However, it has not been demonstrated whether differences in sorting or trafficking are present. Further investigation into this area may also explain why specific neuronal populations (e.g., striatal medium spiny neurons) are particularly vulnerable in neurodegenerative or neuropsychiatric disorders based on trafficking defects.

### The Role of Myosin and BDNF Granule Fusion at the Plasma Membrane

The mechanisms that underlie the final trafficking stages (approach, docking, priming, and fusion) of BDNF-containing secretory granules also remain unclear [51, 82]. Members of the myosin superfamily of motor proteins have been implicated in these stages. Prior studies have demonstrated that secretory granules that are approaching the plasma membrane are transported by myosin Va, myosin 1e, and myosin 1c as part of a multi-motor complex that is regulated by myosin II [82]. Upon arrival at the plasma membrane, the granules are tethered to the actin cytoskeleton by myosin 1c and docked to the membrane via the interaction between myosin Va and the SNARE complex [82]. Finally, the fusion of the docked granule is mediated through myosin VI, myosin II, and myosin 1c [82]. To date, there is no direct evidence linking myosin function to BDNF trafficking. This hypothesis could be tested using live cell imaging of fluorescently tagged BDNF in neurons following siRNA-mediated knockdown of candidate myosins such as myosin Va or VI, which have previously been implicated in secretory granule trafficking. Elucidating these mechanisms may explain what determines the specificity of BDNF vesicle fusion at synaptic versus extrasynaptic sites. While BDNF release has been reported at both sites [124, 125] and plays a key role in synaptic plasticity and circuit function, the exact mechanisms underlying release at each site remain uncharacterized and are an important avenue for future study. Further research regarding these stages could further explain the aberrant secretion of BDNF observed in neurological disorders. For example, the impaired BDNF secretion observed in Huntington’s disease and Alzheimer’s disease may also be due to functional defects in the myosins associated with BDNF-containing secretory granules [82].

## Clinical Implications

The complex structure of neurons makes them especially susceptible to defects in intracellular trafficking, which plays a role in the development of neurological disorders [156]. The sorting, transport, and release of BDNF are essential for its function in neuronal development and synaptic plasticity. Therefore, understanding the mechanisms underlying the trafficking has biomedical significance as impaired BDNF trafficking has been implicated in a wide spectrum of neurodevelopmental, neuropsychiatric, and neurodegenerative conditions. Identifying the molecular machinery that governs BDNF sorting, transport, and release can reveal potential therapeutic targets. Specifically, emerging evidence links altered BDNF trafficking to autism spectrum disorder, schizophrenia, major depressive disorder (MDD), bipolar disorder, Alzheimer’s disease (AD), and Huntington’s disease (HD). Table 3 summarizes trafficking regulators discussed in this review and their associated disease implications.

**Table 3:** Summary of BDNF-trafficking regulators and associated neurodegenerative, neuropsychiatric, and neurodevelopmental disorders

Implicated molecule(s)	Trafficking role	Associated disorder(s)	Evidence summary
**Neurodegenerative disorders**			
**HTT**	Axonal transport	HD	Mutant htt impairs interaction of HAP1 and dynactin to disrupt BDNF transport and impairs interaction of proBDNF with sortilin [17–20] Mutant htt activates axonal JNK3 which phosphorylates kinesin-1 and reduces its binding to microtubules and impairing transport [157]
**CPE**	Sorting	AD	Accumulation of CPE in senile plaques disrupts sorting at the Golgi [21]
**Syt4**	Exocytosis Vesicle targeting	AD HD	Alterations of syt4 levels in patients and transgenic mouse models of AD [158–160] Levels of ARMS, which interacts with syt4, increased, and BDNF secretion is impaired [100]
**Neurodevelopmental disorders**			
**CAPS2**	Exocytosis	Autism spectrum disorder	Mutant CAPS2 impairs axonal release of BDNF [11, 161]
**Neuropsychiatric disorders**			
**BDNF**	Cargo	Schizophrenia MDD	Mutant BDNF results in impaired association with sortilin and HAP1 resulting in retention of BDNF at the Golgi and altered trafficking [9, 15, 16, 18]
**Syt4**	Exocytosis Vesicle targeting	MDD	Alterations in syt4 expression [162, 163] Exposure to chronic unpredictable stress increased expression of syt4 in the medial prefrontal cortex in rodents [164]
**CAPS2**	Exocytosis	Schizophrenia	Levels are altered in patients with schizophrenia [165]
**HTT**	Axonal transport	MDD Schizophrenia	Glucocorticoid signaling increases htt levels and BDNF vesicle transport in cortical neurons [166] Levels are altered in patients with schizophrenia [167]
**KIF13A**	Axonal transport	Schizophrenia	Levels are altered in patients with schizophrenia [167, 168]
**KIF13B**	Dendritic transport		

## Neurodevelopmental and Neuropsychiatric Disorders (Autism Spectrum Disorders, Schizophrenia, and Major Depressive Disorder)

### Autism Spectrum Disorders

Autism spectrum disorders (ASD) are the most diagnosed neurodevelopmental disorder, with patients exhibiting repetitive behaviors and differences in communication and social interaction as compared to the neurotypical population [169]. Given BDNF’s role in development and synaptic plasticity, multiple studies have investigated the relationship of BDNF to ASD. Genomic analyses of patients with ASD have revealed several variations within the *Caps2* gene of some patients [11, 161, 170–177]. For example, a subset of patients exhibited increased expression of the Caps2 isoform *Caps2-dex3*, which lacks a region that binds to dynactin 1, resulting in altered localization of CAPS2 and altered BDNF transport [11]. Furthermore, mice expressing the same variant demonstrated autistic-like behavior (e.g., impaired social behavior, decreased exploratory behavior, increased anxiety) and reduced BDNF release [178]. Similarly, CAPS2 knockout mice exhibit an autistic-like behavior phenotype and impaired activity-dependent BDNF secretion [11]. Taken together, these data not only point to a role for CAPS2 in regulating BDNF secretion, but also demonstrate how dysregulation of CAPS2 results in autism-related behaviors in rodent models and human patients. However, to date, CAPS2 is the only trafficking regulator that has been consistently linked to ASD, and there are no studies that investigate other proteins known to regulate BDNF trafficking and secretion. While previous studies were limited by the lack of relevant human disease models for ASD, patient-derived induced pluripotent stem cells (iPSCs) and organoids, which contain the genetic profile of the original donor, may provide an opportunity to use in vitro models to elucidate how altered BDNF trafficking contributes to ASD pathophysiology [179, 180].

### Schizophrenia and MDD

#### Schizophrenia

Schizophrenia is a highly heritable, chronic, and debilitating neuropsychiatric disorder that affects almost 1% of the general population [181]. This disorder often emerges in early adulthood and is characterized by a broad spectrum of clinical manifestations, including positive symptoms (e.g., hallucinations and delusions), negative symptoms (e.g., anhedonia and blunted affect), and cognitive symptoms (e.g., deficit in memory, attention, and executive function) [182, 183]. The etiology of this disease is complex and reflects the interaction of various environmental and genetic factors [182]. Nevertheless, BDNF may play a significant role in this disorder, as alterations of BDNF levels have been observed in patients diagnosed with schizophrenia [184]. Additionally, the BDNF Val66Met polymorphism has been implicated in schizophrenia. This polymorphism is a naturally occurring human variant of BDNF that results in the amino acid substitution Val66Met in the prodomain of the *bdnf* gene [185, 186]. This substitution impairs the association of BDNF with the sorting receptor sortilin and results in the retention of BDNF at the Golgi and TGN [9]. As a result, there is a decrease in BDNF-containing secretory granules throughout the neuron and an accompanying decrease in activity-dependent BDNF secretion [24, 185, 186]. The BDNF Val66Met polymorphism also affects the association of BDNF with HAP1, a component of the microtubule-associated motor protein complex that facilitates anterograde trafficking [15–18], which further impairs the trafficking of BDNF-containing secretory granules to the plasma membrane [19]. These impairments in BDNF trafficking may alter neuronal morphology and underlie the reductions in hippocampal volume observed in humans with the BDNF Val66Met polymorphism [185]. Liu and colleagues [187] demonstrated that cortical neurons from BDNF Val66Met knock-in mice display dendritic atrophy, a decrease in dendritic spine density, and alterations in dendritic spine morphology. Similarly, in mice where BDNF_Met_ is expressed, there is a significant decrease in the dendritic complexity of neurons from the dentate gyrus [24].

Zhang and colleagues [188] demonstrated that schizophrenic patients carrying the BDNF Val66Met allele display impaired performance in a hippocampus-dependent cognitive task that was accompanied by impairments in attention as compared to schizophrenics carrying the Val allele [188]. In addition to BDNF, several proteins that regulate BDNF trafficking have been implicated in schizophrenia. Notably, alterations in KIF13A and KIF13B expression [167, 168], htt expression [167], and CAPS2, a secretory granule-associated protein implicated in BDNF-regulated exocytosis, have all been noted in patients with schizophrenia [165].

Taken together, these data suggest the possibility that BDNF Val66Met, together with altered CAPS2, htt, and kinesin function, contributes to schizophrenia. However, to date, there are no studies that have directly examined the trafficking of BDNF-containing vesicles in schizophrenia models. This may be because the etiology of schizophrenia is complex and difficult to recapitulate in animal models [183, 189]. Specifically, these models result in the manifestation of some symptoms associated with this disease, but not all. For example, models that use pharmacological methods to induce dopamine system hyperfunction, which has been observed in schizophrenia, exhibit positive symptoms and some cognitive symptoms [183]. Similarly, genetic models of schizophrenia manipulate genes associated with schizophrenia (e.g., DISC-1, dysbindin) individually. However, based on the complexity of schizophrenia, more than one gene is involved [183]. Additionally, genetic manipulations using CRISPR/Cas9 could result in gene mutations that influence animal behavior or result in structural or molecular impairments that could result in difficulty with interpreting results [183]. Finally, it is often difficult to use an animal model to investigate the positive symptoms (e.g., hallucinations, delusions) associated with the disease because animals lack the ability to self-report the presence of these symptoms [190]. Despite these challenges, iPSCs are increasingly being used to investigate the characteristics of schizophrenia, including identification of dysregulated pathways [191], alterations in signaling [192], defects in neuronal morphology [193], defects in neurotransmitter systems [194], and alterations in neuronal connectivity [195]. Future studies should leverage these methods to characterize the extent to which BDNF trafficking is altered in schizophrenia.

#### Major Depressive Disorder

MDD is one of the most common psychiatric illnesses worldwide and is characterized by a depressed mood, persistent anxiety, loss of interest in daily activities, and suicidal ideation [196]. The role of BDNF in MDD has garnered much attention, as previous studies have revealed alterations in serum BDNF levels in patients and rodent models that occur in tandem with upregulation of proinflammatory cytokines, suggesting a role for neuroinflammation [197–200]. Furthermore, similar to schizophrenia, BDNF Val66Met has been implicated in MDD. Specifically, when combined with chronic stress, BDNF Val66Met exacerbates alterations in BDNF levels across brain regions [201, 202].

In addition to BDNF Val66Met, syt4 is proposed to play a role in MDD [164]. Recent studies have revealed alterations in syt4 expression in patients diagnosed with MDD [162, 163]. Furthermore, exposure to chronic unpredictable stress (CUS) (i.e., a paradigm that elicits depressive behaviors) increased expression of syt4 in the medial prefrontal cortex (mPFC) in rodents [164]. Taken together, these data suggest that altered BDNF trafficking contributes to the pathophysiology of MDD. However, the contribution of specific trafficking regulators remains under characterized and represents an important area for future investigation [203]. Furthermore, given the influence of chronic stress and inflammation on BDNF expression, these findings suggest that chronic stress and neuroinflammation may impact the mobilization and release of BDNF-containing vesicles. Consistent with this, exposure to a chronic mild stress paradigm that induces depression altered the trafficking of BDNF mRNA in rodents [204]. Furthermore, *Syt4*^*(−/−)*^ mice exhibit depressive-like behavior [205] while mice exposed to CUS display anhedonia accompanied by increased syt4 expression and reduced BDNF signaling in the mPFC [164].

Interestingly, glucocorticoid (a steroid hormone released by the adrenal cortex in response to stressful stimuli) levels are increased in patients with MDD [206]. Furthermore, Adachi and colleagues [166] demonstrated that increased glucocorticoid signaling in cortical neurons increased levels of htt and, in turn, increased BDNF vesicle transport. Finally, one study demonstrates that the proinflammatory cytokine IL-1β impairs the retrograde transport of BDNF-signaling endosomes in primary culture hippocampal neurons [207]. Collectively, these findings suggest that inflammatory signaling and chronic stress can directly impair BDNF trafficking, which contribute to the pathophysiology of MDD, although further study is needed to elucidate the underlying mechanisms.

## Neurodegenerative Disorders (AD and HD)

### Alzheimer’s Disease

AD is a common, age-related neurodegenerative disorder that is characterized by extensive neuronal loss in the hippocampus and cerebral cortex that manifests as progressive cognitive dysfunction [21, 208]. The role of BDNF in AD pathophysiology has been extensively investigated, as previous studies have demonstrated altered BDNF levels and/or altered BDNF signaling [208] in patients and animal models of AD. The role of BDNF trafficking in this disease has been less extensively studied; however, BDNF-trafficking regulators have been implicated in AD, suggesting that aberrant BDNF trafficking may be a feature of this disease. For example, Plá and colleagues [21] demonstrated that CPE, a sorting receptor that is essential for the targeting of BDNF to the regulated secretory pathway, displays significant accumulation in the senile plaques of Alzheimer’s patients and transgenic mice. Additionally, studies have reported both increase in syt4 levels [158, 159] and decrease in syt4 levels in transgenic mouse models of AD [160]. Furthermore, disrupted axonal transport due to tau hyperphosphorylation and Golgi fragmentation are key components of this disease, suggesting that BDNF sorting at the TGN and subsequent transport could be impaired [209]. Consistent with this, other cargo (e.g., beta-secretase 1 and β-amyloid precursor protein) associated with AD pathophysiology traffic through the RSP, and their transport is altered in AD, further suggesting that altered BDNF trafficking may be indirectly affected [210]. Collectively, while direct evidence is lacking, these findings suggest that the altered sorting, processing, and transport of dense core vesicles containing BDNF may contribute to disease pathogenesis. Therefore, future studies should investigate this possibility as the aberrant transport of BDNF may underlie neuronal loss and cognitive dysfunction observed in AD.

### Huntington’s Disease

HD is a hereditary neurodegenerative disorder resulting from the expansion of a polyglutamine (CAG) repeat in the huntingtin gene [211]. HD is characterized by progressive motor impairment, personality changes, and death within 10–20 years from disease onset. Furthermore, HD results in neuronal dysfunction and death, primarily in the medium spiny projection neurons of the striatum [20]. This loss of medium spiny neurons is due, in part, to altered BDNF transport that results in a reduction of BDNF release [17, 20]. Given the role of BDNF as a prosurvival factor for striatal neurons and the decreased BDNF levels observed in the brains of HD patients, multiple studies have investigated the role of htt in regulating the trafficking of BDNF and the impact of mutant htt on BDNF trafficking [10, 17, 19, 212]. Normal htt interacts with HAP1 to recruit molecular motors and other protein complexes to BDNF-containing secretory granules [17, 20, 68, 213, 214]. However, mutant htt impairs the interaction of HAP1 with other proteins and impairs BDNF trafficking. Specifically, in rodent models of HD and patients with HD, mutant huntingtin impairs axonal BDNF trafficking by disrupting the interaction between HAP1 and dynactin [17, 19, 20]. This disruption results in reduced BDNF release and altered BDNF transport. Similarly, this impairment in BDNF transport and release was observed in striatal neurons [215]. Additionally, this impairment is rescued by the overexpression of wild-type huntingtin [17]. Interestingly, Wu and colleagues [19] demonstrated that mutant htt also impairs the interaction between proBDNF and sortilin, which results in altered BDNF trafficking.

Mutant htt has also been shown to activate axonal JNK3 [157]. In turn, JNK3 phosphorylates kinesin-1, reducing its binding to microtubules and impairing transport [157]. Given that the activation of JNK has been demonstrated to destabilize the interaction of syt4 and KIF1A on BDNF-containing vesicles, these data suggest that mutant htt may activate JNK to reduce BDNF release through syt4 and KIF1A, although to date, this has not been investigated.

ARMS, a neuronal scaffolding protein that interacts with syt4, has also been implicated in HD. Specifically, increased ARMS levels have been observed in HD mouse models and patients with HD [100]. Furthermore, this increase is accompanied by impaired BDNF secretion in mouse models of HD [100]. Finally, in an HD mouse model, the loss of HAP1 resulted in neuronal degeneration in the striatum [216].

Collectively, these studies indicate that altered BDNF trafficking may underlie neuronal loss observed in HD. Specifically, impairments in BDNF sorting, motor recruitment to BDNF-containing vesicles, and BDNF secretion may all work in tandem to induce neuronal loss observed in HD. Previous studies have extensively used rodent models to investigate aberrant trafficking in HD [17–20]. However, iPSCs are increasingly being used to model HD and provide a promising avenue to characterize trafficking defects [217–220]. The use of microfluidic devices that allow cortical neurons to establish functional synapses with striatal dendrites has also recently been demonstrated to recreate the diseased corticostriatal networks observed in HD [221]. Future studies should leverage these methods to further characterize how BDNF transport is impaired in HD.

#### Golgipathies and Coatopathies

Further research that uncovers which adaptor proteins and Arf GTPases regulate BDNF trafficking may also hold clinical significance. Diseases classified as “golgipathies” and “coatopathies” have been used to characterize diseases that arise due to aberrant intracellular trafficking. Golgipathies refer to a group of diseases that, in part, involve defects in the development of the central and/or peripheral nervous system [222]. These defects include epilepsy, brain malformation, intellectual delay, white matter defects, and impaired neurogenesis [222]. Within this group of diseases, mutations in Arf proteins have been identified as an underlying factor. For example, Sakamoto and colleagues [223] characterized de novo variants of the *Arf3* gene in two children who presented with developmental delay, epilepsy, and brain abnormalities. These variants resulted in impaired trafficking from the Golgi [223]. Similarly, Ishida and colleagues [222] characterized a de novo missense Arf1 variant in a patient who also presented with developmental delay. It was observed that the Arf1 variant localized to the Golgi and resulted in increased swelling of the Golgi and an increased recruitment of the coat proteins AP-1 and GGA3. Mutations in coat proteins can also give rise to disorders that are collectively referred to as coatopathies [156]. Of note, > 80% of coatopathies affect the nervous system [156]. For example, mutations in AP-1, AP-2, AP-4, and AP-5 have been demonstrated to give rise to various neurodevelopmental disorders [156, 224, 225]. Similarly, mutations in the coat protein clathrin have been demonstrated to give rise to intellectual disability [226–228, 229, 230, 231]. However, it has not been explored whether these proteins are associated with BDNF trafficking, specifically.

Taken together, these findings implicate BDNF trafficking as a critical point of dysfunction in neurodevelopmental, neuropsychiatric, and neurogenerative disorders. Future studies that further investigate the molecular mechanisms underlying BDNF trafficking may offer new strategies for disease intervention.

## Conclusion

BDNF trafficking in the regulated secretory pathway is a tightly regulated process that involves the action of multiple proteins working in tandem to ensure the proper sorting of BDNF to the RSP, facilitate vesicle maturation, recruit motor proteins, and facilitate exocytosis. While key proteins related to trafficking BDNF via the regulated secretory pathway, such as sortilin, CPE, dynactin, huntingtin, and kinesin, have been identified, multiple molecular players have yet to be identified. Specifically, other proteins that are involved in BDNF sorting and transport, such as granins, adaptor proteins, Arf proteins, and myosins, remain uncharacterized. Given that aberrant trafficking of BDNF has also been implicated in several neurodevelopmental, neuropsychiatric, and neurodegenerative disorders, the investigation of all proteins involved in BDNF trafficking could provide potential therapeutic targets. Clarifying the molecular players of BDNF trafficking is essential for understanding BDNF function as well as identifying areas of dysfunction in disorders marked by impaired BDNF release. Future research should focus on characterizing these proteins through the combined use of quantitative measurement, immunocytochemistry, and live imaging of BDNF trafficking.

## Data Availability

No datasets were generated or analysed during the current study.
